# Manicure

**Published:** 1886-06

**Authors:** 


### Manicure.

There are not nearly as many secrets in manicure as people imagine. A little ammonia or borax in the water you wash your hands with, and that water just lukewarm, will keep the skin clean and soft. A little oatmeal mixed with the water will whiten the hands. Many people use glycerine on their hands when they go to bed, wearing gloves to keep the bedding clean; but glycerine don't agree with every one. It makes some skins harsh and red. These people should rub their hands with dry oatmeal, and wear gloves in bed. The best preparation for the hands at night is white of egg, with a grain of alum dissolved in it. Manicures have a fancy name for it; but all can make it and spread it over their hands, and the job is done. They also make the Roman toilet paste. It is merely white of egg, barley flour, and honey. They say it was used by the Romans in olden times. Anyway, it is a first-rate thing; but it is mean, sticky sort of stuff to use, and don't do the work any better than oatmeal. The roughest and hardest hands can be made soft and white in a month's time by doctoring them a little at bed-time, and all the tools you need are a nail-brush, a bottle of ammonia, a box of powdered borax, and a little fine white sand to rub the stains off, or a cut of lemon, which will do even better, for the acid of the lemon will clean anything. Manicures use acids in the shop, but the lemon is quite as good, and isn't poisonous while the acids are.—*N. Y. Analyst.*

